# Fully endoscopic approach for resection of brainstem cavernous malformations: a systematic review of the literature

**DOI:** 10.1186/s12893-024-02403-5

**Published:** 2024-04-23

**Authors:** Zhigang Hu, Chao Tang, Chiyuan Ma

**Affiliations:** 1 Department of Neurosurgery, Affiliated Jinling Hospital, Medical School of Nanjing University, Nanjing, China; 2 Department of Neurosurgery, Jinling Hospital, Southern Medical University, Nanjing, Jiangsu China; 3 Department of Neurosurgery, Jinling Hospital, Nanjing Medical University, Nanjing, 210002 China

**Keywords:** Endoscopic, Surgery, Brainstem, Cavernous malformations

## Abstract

**Background:**

Brainstem cavernous malformations (BCMs) are benign lesions that typically have an acute onset and are associated with a high rate of morbidity. The selection of the optimal surgical approach is crucial for obtaining favorable outcomes, considering the different anatomical locations of various brainstem lesions. Endoscopic surgery is increasingly utilized in treating of BCMs, owing to its depth illumination and panoramic view capabilities. For intra-axial ventral BCMs, the best surgical options are endoscopic endonasal approaches, following the “two-point method. For cavernous hemangiomas on the dorsal side of the brainstem, endoscopy proves valuable by providing enhanced visualization of the operative field and minimizing the need for brain retraction.

**Methods:**

In this review, we gathered data on the fully endoscopic approach for the resection of BCMs, and outlined technical notes and tips. Total of 15 articles were included in this review. The endoscopic endonasal approach was utilized in 19 patients, and the endoscopic transcranial approach was performed in 3 patients.

**Results:**

The overall resection rate was 81.8% (18/22). Among the 19 cases of endoscopic endonasal surgery, postoperative cerebrospinal fluid (CSF) leakage occurred in 5 cases, with lesions exceeding 2 cm in diameter in 3 patients with postoperative CSF rhinorrhea. Among the 20 patients with follow-up data, 2 showed no significant improvement after surgery, whereas the remaining 18 patients showed significant improvement compared to their admission symptoms.

**Conclusions:**

This systematic literature review demonstrates that a fully endoscopic approach is a safe and effective option for the resection of BCMs. Further, it can be considered an alternative to conventional craniotomy, particularly when managed by a neurosurgical team with extensive experience in endoscopic surgery, addressing these challenging lesions.

**Supplementary Information:**

The online version contains supplementary material available at 10.1186/s12893-024-02403-5.

## Background

The brainstem, located between the cerebral hemispheres and the spinal cord, is a crucial component of the central nervous system. It plays a fundamental role in vital functions including respiration, cardiovascular regulation, and consciousness [1]. The brainstem consists of distinct structures such as the midbrain, pons, and medulla. The midbrain is involved in visual processing, eye movements, and auditory processing. The pons controls motor control and sensation from the face. The medulla controls vital functions such as heart rate, respiration, and blood pressure. Further, the brainstem serves as a crucial intersection for neural projections between the cerebral cortex, basal ganglia, thalamus, cerebellum, and spinal cord. Additionally, brainstem nuclei fine-tune or regulate neural circuits. Specialized cores within the brainstem, such as the locus coeruleus and raphe nuclei, extensively project to other subcortical brain structures. This projection influences their functions by modifying local neurotransmitter levels and maintaining homeostasis [2].

In addition to its role in physiological functions, the brainstem plays a crucial role in consciousness and alertness [3]. Lesions or damage to the brainstem can result in coma or altered levels of consciousness due to the interruption of neural pathways that control consciousness. Therefore, it is essential to preserve the integrity of the brainstem during surgical interventions to avoid potential neurological deficits or even loss of consciousness.

Brainstem cavernous malformations (BCMs) are rare, benign vascular lesions with a high tendency for symptomatic bleeding, estimated at approximately 6% per year [4]. In BCM bleeding cases, the re-bleeding rate is 5 to 35% per year [5, 6]. Surgical intervention is recommended for patients with BCM two or more symptomatic hemorrhages [7]. The choice of surgical approach depends on the location of the cavernous malformations within the brainstem. The main goal is to minimize damage to healthy tissue and achieve complete excision.

Historically, BCMs have been resected using various skull-based microsurgical approaches based on the two‑point method [8]. The morbidity associated with these approaches is primarily related to the damage caused when inflicted resulting from entering the dorsal brainstem to resect intra-axial or ventral lesions. Consequently, the management of BCM is challenging, and the risk of a high surgical incidence should be carefully assessed.

In the resection of BCMs, we amid to select an approach that provides the identification of convenient entry points to the brainstem, fully displays the entire lesion, and provides the necessary working angle for gross total excision, minimizing disruption to the adjacent parenchyma. Recent study has reported that endoscopic approaches to BCMs outperform traditional standard operating microscope in minimizing trauma and improving visualization [9].

This manuscript presents a systematic review of the literature to assess the feasibility of endoscopy in the management of BCMs, and discusses its advantages, limitations and technical notes.

## Patients and methods

A PubMed search was performed to identify endoscopic approaches for BCMs between 2012 and 2024. We formulated our search strategy using different combinations of keywords, including “surgery,”, “cavernoma,” “cavernous malformation,” “cavernous angioma,” and “brainstem”. Titles and abstracts were reviewed to identify cases of BCM treated using a fully endoscopic approach. Additionally, the reference lists of the selected articles were manually searched to identify additional studies.

Exclusion criteria included cadaveric or radiological studies and combined case data. Several variables in each study were quantified, including the number of patients, sex, age, lesion location, lesion size, symptoms, use of neuronavigation, cerebrospinal fluid (CSF) drainage, CSF leak, surgical approach, degree of resection, postoperative complications, duration of follow-up, and prognosis.

This manuscript presents a systematic review of the literature on endoscopic endonasal surgery for BCMs, applying the Preferred Reporting Items for Systematic reviews and Meta-Analyses (PRISMA) guidelines.

## Results

Fourteen studies met the inclusion criteria and were included in the this review (Table 1). study participants consisted of 16 females (72.7%) and 6 males (27.3%), with a mean age of was 36.1 years (range: 14–64 years). The lesion localization was predominantly pontine (15 cases, 68.2%), followed by the midbrain (4 cases, 18.2%), cervicomedullary junction (1 case, 4.5%), junction between the posterior mesencephalon and the upper pons (1 case, 4.5%), and medulla oblongata (1 case, 4.5%). Data on mass dimensions were available for 18 of the 22 cases; the maximum diameter of the tumor was > 2 cm in 10 cases and ≤ 2 cm in 8 cases. Total lesion resection was performed in 10 cases with a maximum diameter >2 cm, and in 6 cases with a maximum diameter ≤ 2 cm. The main symptoms reported included in 5 patients (22.7%), diplopia in 10 (45.5%), hemiparesis in 12 (54.5%), facial nerve deficit in 5 (22.7%), dysphagia in 3 (13.6%), and nausea and vomiting in 2 (9.1%). Additionally, coma, dysarthria, dysphonia, and cranial nerve III palsy was each present in 1 patient (4.5%).

**Table 1:** clinical cases of fully endoscopic approach for resection of brainstem cavernous malformations

Study	Gender	Age	Lesion location	Lesion size	Symptom	Navigation	Surgery approach	Postoperative MRI	CSF drainage	CSF Rhinorrhea	Re-intervention	Complication	Prognosis	Follow-up
Kimball et al., 2012	female	59	the ventral midpons eccentric to the righ	2.3 × 2.2 × 2.5 cm	bilateral facial numbness, diplopia, and complete left hemiplegia	Yes	endoscopic endonasa transclival	total	No	Yes	Yes	a right cranial nerve VI and peripheral cranial nerve VII palsy and left-sided hemiplegia	improve	1 month
Sanborn et al., 2012	male	17	ventromedial pons, eccentric to the right side	1.7 × 1.2 cm	a left-side hemiparesis, a right sixth nerve palsy, and dysphagia	Yes	endoscopic endonasa transclival	total	Yes	Yes	Yes	left-side hemiparesis, vertical nystagmus, right-side facial weakness, and bilaterally restricted horizontal eye movements on both left and right gaze	improve	6 months
Enseñat et al., 2015	male	NA	a ventral midline mesencephalon	NA	third cranial nerve palsy	No	endosendoscopic transnasal transtuberculum-transplanum approach	subtotal	No	No	No	None	improve	NA
Nayak, et al., 2015	female	39	the left dorsal midbrain	1.8 × 1.3 cm	headache, double vision, and rightsided numbness	No	endoscopic retrosigmoid craniotomy	total	No	No	No	None	improve	12 months
	female	59	the left dorsal pons	1.8 × 1.4 cm	left ear hearing loss and facial paresis	No	endoscopic retrosigmoid craniotomy	subtotal	No	No	No	facial paralysis(6/6)	same	18 months
	female	60	the ventromedial cervicomedullary junction	0.8 × 0. 9 × 1 cm	right hemiparesis	Yes	endoscopic endonasa transclival	total	No	No	No	None	same	3 months
	male	17	in the ventromedial pons	2.1 × 1.7 cm	headache and rightsided facial numbness	Yes	endoscopic endonasa transclival	total	No	Yes	Yes	left hemiparesis and rightsided, vertical nystagmus and restricted horizontal gaze bilaterally facial weakness	improve	24 months
Linsler et al., 2015	female	29	the ventral pons	2.0 × 1.8 × 2.2 cm	Numbness and tingling of right arm and leg, loss of fine motor control of right hand, transient diplopia, transient headache	Yes	endoscopic endonasa transclival	total	Yes	No	No	None	improve	6 weeks
Dallan et al., 2015	male	15	ventromedial pons, eccentric to the right side	1.0 × 1.0 cm	severe cephalalgia with vomiting and neurological signs including diplopia, sudden hearing loss and facial palsy	Yes	endoscopic endonasa transclival	subtotal	No	No	No	None	improve	24 months
Shi-Ming He et al., 2016	female	20	the right ventromedial mesencephalon	1.2 × 1.7 cm	left-sided hemiparesis, restriction of medial and lateral left-eye movements, and loss of left pupillary light reflex	Yes	endoscopic endonasa transclival	total	Yes	No	No	None	improve	3 months
Gómez-Amador JL et al., 2017	male	29	ventral pons	1.8 × 2.6 × 2.9 cm	Acute occipital headache, nausea, horizontal diplopia. Somnolence, facial palsy, dysarthria, dysphonia, dysphagia, left hemiparesis	Yes	endoscopic endonasa transclival	total	No	No	No	None	improve	5 months
Puya Alikhani et al., 2019	female	26	left medulla oblongata	1.5 cm	imbalance, swallowing difficulty, and right hemibody weakness	Yes	endoscopic endonasa transclival	total	No	No	No	None	improve	3 months
Xiao Dong et al., 2021	female	28	a ventral pontine lesion eccentric to the left	2.3 × 2.0 cm	right-sided hemiparesis, diplopia and hemiparethesia	Yes	endoscopic endonasa transclival	total	Yes	No	No	None	improve	1 month
Lima et al., 2019	female	46	pons	2.6 cm	mild right hemiparesis	No	endoscopic endonasa transclival	total	No	Yes	Yes	a slight worsening of right hemiparesis	improve	6 months
Cecchini, 2019	female	40	the junction between posterior mesencephalon and upper pons	NA	None	Yes	endoscopic subtemporal approach	total	Yes	No	No	None	same	NA
Goldschmidt et al., 2019	female	25	midbrain	NA	diplopia and headache	Yes	endoscopic endonasa transclival	total	Yes	No	No	None	improve	1 month
Paolo Priore et al., 2022	female	14	ventral pons	NA	a state of coma	Yes	endoscopic endonasa transclival	subtotal	No	No	No	None	improve	96 months
Takeuchi K et al., 2023	female	64	ventral pons	2.3 × 2.3 × 2.5 cm	Lt hemiparesis 2/5, CN VI palsy, mRS 4	NA	endoscopic endonasa transclival	total	No	No	No	None	improve	3 months
	female	44	ventral pons	3.5 × 3.4 × 3.8 cm	Lt hemiparesis 2/5, mRS 4	NA	endoscopic endonasa transclival	total	No	No	No	None	improve	3 months
	female	33	ventral pons	1.6 × 1.2 × 1.8 cm	Lt hemiparesis 4/5, CN VI palsy, mRS 3	NA	endoscopic endonasa transclival	total	No	Yes	Yes	None	improve	3 months
	female	49	ventral pons	1.6 × 1.6 × 2.5 cm	Lt hemiparesis 4/5, CN VI palsy, mRS 3	NA	endoscopic endonasa transclival	total	No	No	No	Transient worsening of CN VI palsy	improve	3 months
	male	46	ventral pons	2.0 × 2.4 × 2.0 cm	Rt hemiparesis 4/5, mRS 3	NA	endoscopic endonasa transclival	total	No	No	No	None	improve	3 months
NA: No data; CSF: Cerebrospinal fluid; LT: left; Rt: right; mRS: modified Rankin Scale														

NA: No data. CSF: Cerebrospinal fluid

In a cohort of 22 patients, the data indicated intraoperative neuronavigation in 13. Among the surgical approaches employed, an endoscopic endonasal transclival approach was used in 18 patients and endoscopic transnasal transtuberculum-transplanum approach was performed in 1 patient. The remaining three patients underwent endoscopic lateral supracerebellarinfratentorial (SCIT), endoscopic retrosigmoid craniotomy and endoscopic subtemporal approaches, respectively. Among the 19 patients undergoing endoscopic transnasal surgery, postoperative Magnetic Resonance Imaging (MRI) showed residual lesions in 4 cases, and the remaining 15 patients were completely resected. A lesion primarily located in the left dorsal midbrain was completely resected via a retrosigmoid craniotomy using an endoscopic SCIT approach. A lesion in the left posterolateral pons adjacent to the cranial nerve VII/VIII complex was subtotally excised using a retrosigmoid approach. Lastly, a lesion at the junction between the posterior mesencephalon and the upper pons was completely excised using a fully endoscopic subtemporal approach.

In the 19 cases of endoscopic endonasal surgery, data indicated that postoperative CSF leakage in 5 cases. Notably, lumbar drainage was performed in 3 of these cases after surgery; however, all 5 patients experienced CSF leakage requiring surgical revision. Among the 5 patients with postoperative CSF rhinorrhea, the maximum lesion diameter exceeded 2 cm in 3 cases. Among the entire cohort of 22 patients, postoperative complications occurred in six patients, including cranial nerve VI and VII palsy, hemiparesis, vertical nystagmus and facial paralysis. Among the 20 patients with available follow-up duration (range:1–96 months), three patients did not experience significant improvement after surgery; Conversely, the remaining 13 patients showed significant improvement during follow up, compared to their admission symptoms.

### Endoscopic endonasal approach

#### Endoscopic endonasa transclival approach

**Fig. 1:**
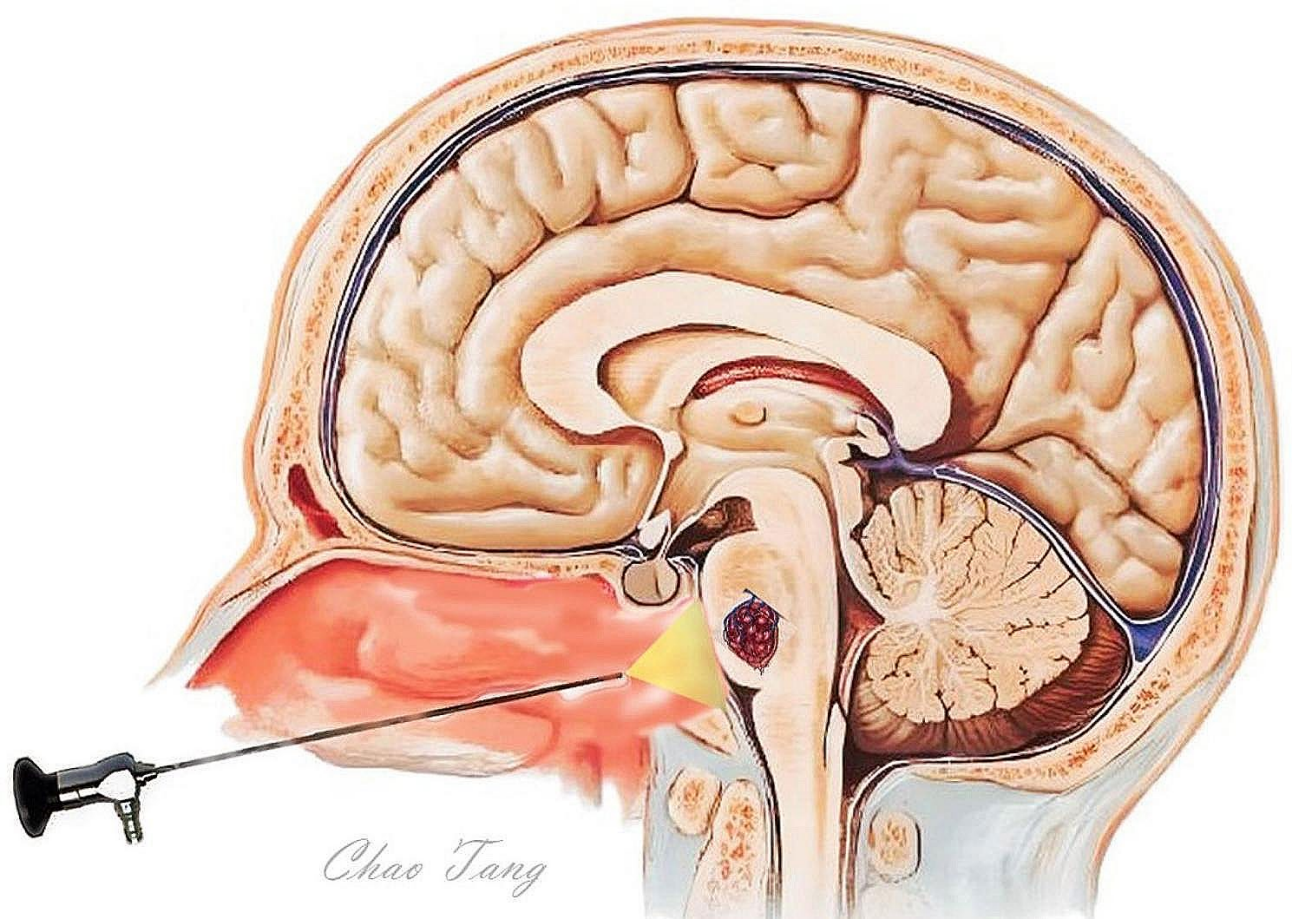
Endoscopic endonasa transclival approach for ventral pontine cavernous malformations. The anterior face of the sphenoid sinus was removed to expose the clivus; The clival recess and the junction of the pons and pontomedullary junction were the superior and inferior limit of the bony exposure

Preoperative MR–diffusion tensor imaging (MR-DTI) is useful for establishing a surgical plan, especially for ventral brainstem lesions. Intraoperative neuronavigation and neurophysiological monitoring are recommended. The bilateral nasal approach and two-surgeon technique were used with and without endoscopic holders. The middle turbinate was lateral displaced, and the right nasoseptal flap was dissected following the sphenopalatine artery. To create adequate operative space, a partial middle turbinectomy and removal of the posterior half of the nasal septum were performed. The anterior wall and floor of the sphenoid sinus were removed to expose the clival region. The clivus was then drilled down to the clival dura at the midline. The bone on the paraclival carotid arteries usually does not require extensive removal. The dura was opened along the midline. A small incision was made in the overlying pia lesion with assistance from neuronavigation. Lesion resection was performed using gentle suction and sharp dissection. A 30°or 45° endoscope was used to inspect the cavity and ensure complete resection of the cavernoma. The surgical cavity was covered with an oxidized cellulose hemostatic agent. For postoperative reconstruction, we recommend a meta-analysis of the strategies for skull base reconstruction [10]. Endoscopic endonasa transclival approach to ventral pontine cavernous malformations is shown in Fig. 1.

#### Endosendoscopic transnasal transtuberculum-transplanum approach

**Fig. 2:**
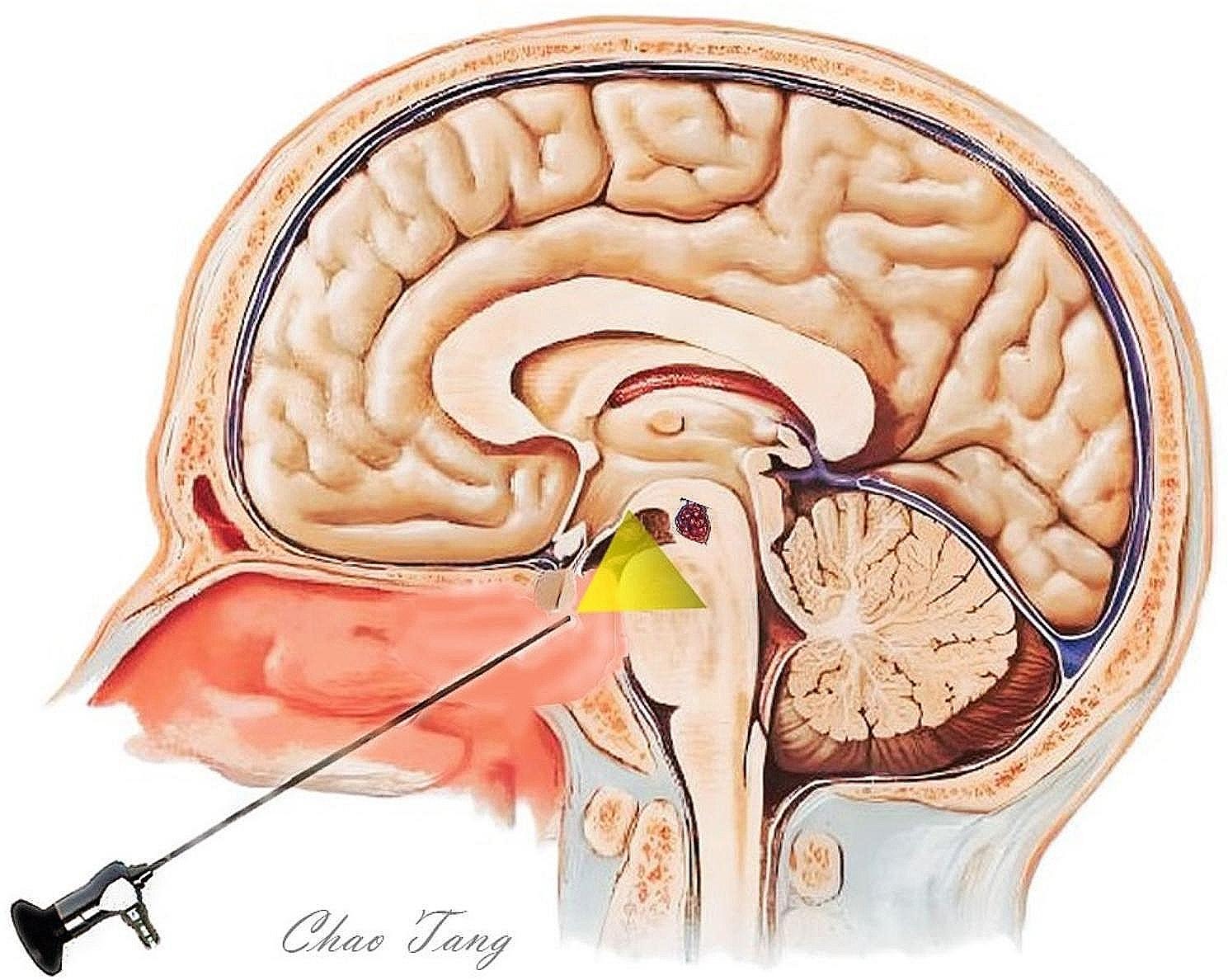
Endosendoscopic transnasal transtuberculum-transplanum approach to ventral mesencephalic cavernous malformations. The sellar floor and the upper clivus were the superior and inferior limit of the bony exposure; Pituitary transposition is used to increase access to lesions behind the upper clivus

The endoscopic transnasal transtuberculum-transplanum approach is the straightest and safest route for accessing cavernoma surfaces in the ventral mesencephalon. Utilizing the extradural pituitary transposition technique increase the access to lesions located behind the clivus. This approach provides greater access to the upper clivus and reduces the risk of pituitary dysfunction. Typically, these lesions were found between the mesencephalon and the basilar artery, positioned inferior to the bilateral mammillary bodies, posterior communicating arteries, and perforators. Critical structures such as the perforator and venous structures of the midbrain should be carefully identified during surgery to minimize the risk of injury. The tumor was removed by gentle suction and careful dissection. Endosendoscopic transnasal transtuberculum-transplanum approach to ventral mesencephalic cavernous malformations is shown in Fig. 2.

## Discussion

BCMs is a relatively rare condition, accounting for 8–16% of all cavernous malformations in the brain [11, 12]. These vascular malformations involve abnormal blood vessels in the brainstem. Multistage bleeding within the sinusoid structure of BCMs results in their expansion into a mulberry-like structure, potentially causing several adverse effects on the brain [13]. They can cause a variety of symptoms, including paralysis, weakness, and difficulty speaking, and in severe cases, even death. The complex anatomy of the brainstem, couple with the presence of key cranial nerve nuclei, renders surgical intervention for BCMs a highly challenging task. Ideally, this approach should minimize the traversal of normal brain tissue before reaching the lesion. The principle of the two-point method in BCMs surgery involves drawing a line from the center of the lesion to the point closest to the brain surface [14]. Although this approach is a good starting point, BCMs present unique challenges that require modifications to the final approach.

Surgical resection is required in patients with hemorrhagic onset, progressive neurological symptoms, and cavernous malformations that extend very close to the pial or ependymal layer. The surgical treatment of BCMs is a highly specialized procedure that should only be performed by experienced neurosurgeons. Despite its complexity and associated risks,, surgery can offer significant benefits to patients in terms of both survival and quality of life. Dorsal midbrain cavernous malformations are particularly challenging to address because of their deep location within the brain. These lesions are typically resected through the unilateral superior and inferior colliculi using an occipital transtentorial approach. Ventral midbrain cavernous malformations are usually resected through the cerebral peduncle between the corticospinal tract and origin of the oculomotor nerve, using a pterional or orbitozygomatic approach. Most pontine cavernous malformations are resected through the unilateral supra-facial triangle on the floor of the fourth ventricle, using the telovelar approach. Some lateral pontine lesions were treated with a lateral suboccipital craniotomy and excised through the lateral pontine between the origins of the facial and glossopharyngeal nerves. A posterior transpetrosal approach was used to remove ventral pontine cavernous malformations between the origins of the trigeminal and vestibulocochlear nerve initiation points. Medullary BCMs were excised using a suboccipital midline craniotomy through the dorsal medulla around the olive or through the dorsal midline of the medulla caudal to the obex [15]. Several studies have been published regarding the use of different surgical approaches for treating BCMs. Spetzler was the first to describe “minimization” of surgical approaches for BCMs by abandoning skull base approaches with the highest morbidity, such as transpetrosal and subtemporal approaches [8, 16, 17]. Mai et al. described a minimally invasive resection technique using diffusion tensor imaging to study distortion of the underlying feasibility. In such cases, the cavernous hemangioma is usually removed internally to decompress the lesion, and the wall is gently removed from the surrounding brainstem and disconnected using a bimanual technique [4]. Although effective in most patients with BCMs, this method has drawbacks, such as a small surgical window, and challenges in achieving total resection.

Given these challenges associated with surgical resection of BCM, the use of endoscopes emerges as a valuable tool. Endoscope complement minimally invasive resection techniques described above through small operative window. Unlike traditional operating microscopes, endoscopes do not require a large workspace. They can improve deep illumination and provide a panoramic view of the surgical cavity. Anatomical studies have demonstrated the utility of endoscope-assisted approach in traditional open surgical approaches [18, 19].

A recent comprehensive analysis of endoscopically-assisted resection of BCMs established endoscopy as a highly effective adjunct in the surgical management of these lesions. Among the 19 patients who underwent 20 surgical procedures, resection of BCMs was performed under microscopic guidance. Periodic endoscopic inspection was utilized to supplement visualization in all cases except one, where the transsphenoidal approach was executed solely by endoscopy [20]. In this review, we amid to explore the potential benefits of purely neuroendoscopic techniques using different approaches for treating BCMs.

### Fully endoscopic techniques in surgery for the ventral lesion of brainstem

In the current review, data from 19 patients with brainstem ventral cavernous hemangiomas were collected. The use of the endoscopic transnasal transclival approach for the treatment of ventral pons cavernomas has been reported [9, 21–29]. In cases of cavernous malformations in the ventral pons, the shortest distance between lesion and the normal brainstem is undoubtedly a direct ventral approach. The transoral translabial approach was previously proposed in a report of two successfully treated cases to minimize intraoperative trauma to eloquent neural structures and morbidity [30]. However, a large transoral series has shown that this approach is associated with significant mortality [31]. It has been observed that three patients who underwent endoscopic transnasal surgery experienced subtotal resection of the ventral BCMs in this review. The presence of residual lesions may be due to various factors such as the location, size, and invasiveness of the lesion, as well as the surgical technique and experience of the surgeon. In one patient, the core of the cavernous hemangioma was difficult to identify because of intrapontine bleeding, resulting in subtotal resection. No post-operative CSF leakage was observed in this patient, and a neurological examination two months after surgery revealed significant improvement in the palsy/dysfunction of cranial nerves VI, VII, and VIII [26]. Another patient who underwent subtotal resection had no other adjuvant therapies and remained stable over a follow-up period of 8 years [21]. In addition, owing to the anatomical orientation, it was too risky to attempt any other surgical maneuvers, resulting in subtotal resection of the lesion [32]. It was reported that MR-DTI and direct brainstem cortical stimulation were used in endoscopic endonasal transclival approach for ventral pontine cavernous hemangiomas to help ascertain the proximity of the corticospinal tract (CST) to the lesion [29]. It is noteworthy that all lesions were successfully completely resected in five cases, and the symptoms of hemiplegia improved after surgery. The success of the surgery can be attributed to the preoperative determination of the positional relationship between the CST and BCM, endoscopic technique and intraoperative neuromonitoring. Direct cortical and subcortical stimulation is particularly crucial during the removal of tumors adjacent to or involving the motor cortex and CST. The combination of MR-DTI and neuronavigation techniques in surgical cases in Gomez-Amador et al. and He et al. also achieved satisfactory therapeutic results [24, 33].

Surgeons must weigh the advantages of direct access to the lesion, minimizing surgical trauma, the known risk of CSF leakage, and the possible use of endoscopic instruments to deal with bleeding. In this review, only five cases of postoperative CSF leaks were encountered. In three of the five patients with postoperative CSF rhinorrhea, the maximum lesion diameter > 2 cm. This is understandable because a larger lesion requires greater exposure of the skull base, leading to a higher risk of postoperative CSF leakage. One patient presented with CSF rhinorrhea 26 days after endoscopic surgery; a new fat graft was harvested and placed into the defect, the nasoseptal flap was replaced with fibrin glue and Avitene, and CSF drainage was continued for 5 days postoperatively through a right side ventriculostomy [27]. Nayak et al. described a similar case and the management of CSF rhinorrhea after surgery [9]. The CSF rhinorrhea occurred on the 5th postoperative day when the Foley catheter was removed, after the lumbar drainage failed, the same reconstruction strategy was re-performed, the patient suffered no further CSF leakage [28]. The patient presented with a CSF leakage on the 6th postoperative day, the reconstruction layers were repositioned and fixed with fibrin glue and a Foley catheter, and lumbar drainage was performed [23]. Postoperative CSF leakage was observed in one of five patients in Takeuchi K’s report, which was resolved by replacing the abdominal subcutaneous fat graft and overlaying it with a nasoseptal flap [29]. Skull base reconstruction to avoid postoperative CSF leakage is a well-described and standardized technique that varies little among surgeons [34]. Our meta-analysis of reconstruction strategies for intraoperative CSF leakage in endoscopic endonasal skull base surgery showed that mucosal flap and inlay for high-flow intraoperative CSF leakage and tampon (compared with balloon) for low-flow intraoperative CSF leakage, improved the postoperative CSF leakage rate [10].

Three reports described the endoscopic approaches to the midbrain region of the brainstem [32, 33, 35]. The anatomical location of the midbrain is higher than that of the pons; to reach the surgical area, the sella, tuberculum, and planum need to be removed. Enseñat et al. described an endoscopic transnasal transplanum-transtuberculum approach to the ventral midline mesencephalon, in which a small portion of the normal pituitary gland was removed to improve surgical access. After surgery, the symptoms of oculomotor nerve paralysis were completely eliminated, and no new neurological or endocrine abnormalities occurred [32]. He [33] described a right ventromedial mesencephalon lesion approached via the endoscopic transnasal transtuberculum-transclival route. In this case, the surgical channel passed below the pituitary gland and transdural transposition was performed to expand the surgical field. Thus, direct manipulation of the gland was avoided, thereby reducing the risk of hypopituitarism. Interestingly, considering the differences between the two surgical approaches, the transtuberculum-transclival approach resulted in complete resection of the lesion, whereas the transplanum-transtuberculum approach resulted in a subtotal resection. Therefore, even in the presence of extension to the third ventricle, the transtuberculum-transclival approach seems to be a better choice for treating mesencephalic cavernous hemangiomas. The middle and lower parts of the brainstem are located behind the clivus and treated naturally using a transclival approach. However, this patient still required semi-translocation of the pituitary gland to access this midbrain area [35]. The patient underwent postoperative lumbar drainage, and no CSF rhinorrhea, total tumor resection, or neurological dysfunction was observed. In this review, there were two cases in the medulla oblongata, it is important that the bone from the middle of the clivus down to the C1 ring needs to be removed, and the underlying dura was exposed [9, 36].

### Fully endoscopic techniques in surgery for the dorsal and lateral lesion of brainstem

Microsurgical approaches such as SCIT, retrosigmoid craniotomy, and subtemporal approaches have been widely used to treat cavernous hemangiomas in the dorsal or lateral regions of the brainstem [5]. Nayak et al. described an endoscopic SCIT approach for left dorsal midbrain lesion, and an endoscopic retrosigmoid craniotomy approach for lateral pontine lesion, which also demonstrated good results [9]. A 39-year-old female patient underwent a surgical procedure in the lateral position, where a large sigmoid incision was made behind her ear, followed by a 3 cm craniotomy performed in the sigmoid region. The endoscope was stabilized by the Mitaka pneumatic holding arm, which provided an exceptional view and was used as the sole magnification tool for resection. Standard bimanual techniques were employed to dissect arachnoid adhesions, while standard microsurgical instruments were used to remove the BCM. A 59-year-old woman with left pontine CM underwent a similar endoscopic surgical procedure. The lesion was resected until normal brainstem tissue was obtained. However, MRI performed at the 6-month follow-up showed residual BCM in the superior aspect of the cavity. Cecchini recently described the benefits of an endoscopy for junction lesion between the posterior mesencephalon and the upper pons through a subtemporal approach [37]. In the surgical procedure detailed in the case report, a small incision was made above the ear and a 2.5 cm craniotomy was performed near the floor of the middle cranial fossa on the temporal squama. After dural opening, a four-handed endoscopic technique was used for dissection. During the dissection process, suction and grasping microforceps were used to stabilize a cavernous malformation, which was then detached from the brainstem parenchyma.

### Limitation

Although endoscopes can improve depth illumination and panoramic views, the two-dimensional visualization of the surgical field provided by endoscopes compared to microscopes may lead to disorientation for spatially inexperienced surgeons. In particular, for ventral brainstem lesion, the narrow endonasal surgical corridor may be challenging, and if important arterial vessels are damaged, the results can be catastrophic. Therefore, patients should be carefully selected at the onset of endoscopic endonasal surgery to avoid complications. In addition, endoscopic endonasal approaches to the brainstem is at risk of invading the motor tracts, which may lead to the additional neurological dysfunction. Careful evaluation of the nerve tract should be carried out through techniques such as high-definition fiber tracking to reduce this risk [38]. Compared with microsurgery, postoperative CSF leakage remains a high-risk factor for endoscopic endonasal surgery.

## Conclusions

Endoscopic endonasal skull base surgery for BCMs is a controversial topic in the neurosurgical practice. However, this systematic literature review, demonstrates that a fully endoscopic endonasal transclival approach to the ventral surface lesions of the brainstem is a safe and effective option for resection. BCMs located along the dorsal and lateral sides may also be considered valid options for endoscopic resection due to the panoramic view when compared with open transcranial approaches. Postoperative CSF leakage is the most common complication and an important cause of morbidity. Multilevel reconstruction should be considered in every case. The incidence of CSF leakage after endoscopic transnasal resection of lesions less than 2 cm in diameter appears to be lower. This may be a condition for carefully selected cases of resection via endoscopic endonasal surgery. Additionally, MR-DTI and direct brainstem cortical stimulation are instrumental in determining the proximity of the CST to the CM. The endoscopic endonasal transclival approach offers a direct route to the lesion, making it a safer treatment option for patients with CST courses laterally or posteriorly to BCMs. Currently, only limited literature exists on endoscopic endonasal approaches for BCMs, and no prospective randomized controlled study has been conducted to definitively determine the therapeutic role of endoscopy.

### Electronic supplementary material

Below is the link to the electronic supplementary material.

Supplementary Material 1

## Data Availability

The datasets used and/or analysed during the current study available from the corresponding author on reasonable request.

## Abbreviations

BCMs: Brainstem cavernous malformations
CSF: cerebrospinal fluid
SCIT: supracerebellarinfratentorial
MRI: Magnetic Resonance Imaging
MR-DTI: MR–diffusion tensor imaging
CST: corticospinal tract
