# Supplementary material for: Fully endoscopic approach for resection of brainstem cavernous malformations: a systematic review of the literature
Source: BMC Surg. 2024 Apr 23;24:120. doi: 10.1186/s12893-024-02403-5 (PMC11036755; doi:10.1186/s12893-024-02403-5)
Supplement: Supplementary file 1 — Supplementary Material 1 [file 12893_2024_2403_MOESM1_ESM.docx]

**Identification of studies via databases and registers**

Records removed *before screening*:

Duplicate records removed (n = 4)

Records marked as ineligible by automation tools (n =0 )

Records removed for other reasons (n =2 )

Records identified from*:

Databases (n =22 )

Registers (n =0 )

**Identification**

Records screened

(n = 16)

Records excluded**

(n = 0)

Reports sought for retrieval

(n =2 )

Reports not retrieved

(n =2 )

**Screening**

Reports assessed for eligibility

(n = 17)

Reports excluded:

Reason Endoscope-assisted resection (n =2 )

Studies included in review

(n =14 )

Reports of included studies

(n = 17)

**Included**

*Consider, if feasible to do so, reporting the number of records identified from each database or register searched (rather than the total number across all databases/registers).

**If automation tools were used, indicate how many records were excluded by a human and how many were excluded by automation tools.

*From:*  Page MJ, McKenzie JE, Bossuyt PM, Boutron I, Hoffmann TC, Mulrow CD, et al. The PRISMA 2020 statement: an updated guideline for reporting systematic reviews. BMJ 2021;372:n71. doi: 10.1136/bmj.n71

For more information, visit: <http://www.prisma-statement.org/>
